# A review on chloride induced corrosion in reinforced concrete structures: lab and *in situ* investigation

**DOI:** 10.1039/d4ra05506c

**Published:** 2024-11-21

**Authors:** Mohsin Ali, Muhammad Alamgeer Shams, Naraindas Bheel, Abdulrazak H. Almaliki, Abubakar Sadiq Mahmoud, Yakubu Aminu Dodo, Omrane Benjeddou

**Affiliations:** a Graduate School of Urban Innovation, Department of Civil Engineering, Yokohama National University Kanagawa 240-8501 Japan; b Department of Civil Engineering, University of Engineering and Technology Lahore Pakistan malamgeershams@yahoo.com; c Department of Civil and Environmental Engineering, Universiti Teknologi PETRONAS Bandar Seri Iskandar Tronoh Perak 32610 Malaysia; d Department of Civil Engineering, College of Engineering, Taif University Taif 21944 Saudi Arabia; e Interdisciplinary Research Center for Construction and Building Materials, King Fahd University of Petroleum and Minerals 31261 Dhahran Saudi Arabia; f Safety Technology, Dammam Community College, King Fahd University of Petroleum & Minerals Dhahran 3126 Saudi Arabia; g Architectural Engineering Department, College of Engineering, Najran University 66426 Najran Saudi Arabia; h Department of Civil Engineering, College of Engineering, Prince Sattam Bin Abdulaziz University Alkharj 16273 Saudi Arabia

## Abstract

Reinforced concrete (RC) constructions are seriously threatened by chloride-induced corrosion (CIC) and carbonation, which can result in structural degradation, safety issues, and financial losses. Electrochemical methods and microstructural analysis tests are some of the laboratory techniques used to examine key elements of CIC, such as the impact of different variables and the efficacy of mitigation solutions. *In situ* studies that make use of non-destructive testing, chloride profiling, and half-cell potential measurements offer important new insights into the long-term performance and causes of RC structure deterioration in real-world circumstances. Non-destructive approaches for CIC detection are emerging these days and provide fruitful results. Studies have focused on the use of these approaches for CIC detection on small specimens in the lab as well as on full-scale experiments in the field. This review covers both *in situ* monitoring and laboratory studies to provide a thorough analysis of CIC.

## Abstract 2

Advanced tools like 3D laser scanning, X-ray μCT, and optical fiber sensing now outperform traditional methods like HCP and resistivity tests, which often fail to detect chloride-induced corrosion accurately.

## Graphical abstract

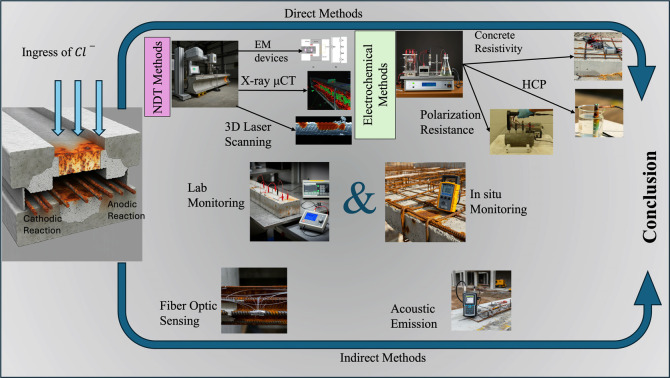

## Introduction

The most utilized structural material for modern infrastructure and buildings is reinforced concrete (RC). There are numerous benefits to using steel and concrete together. Steel makes up for concrete's low tensile strength, and the alkaline concrete pore solution promotes the development of a passive coating that guards the steel from corrosion. Under typical conditions, RC structures operate well for the duration of their designed service life. However, chloride penetration and/or concrete carbonation might destroy the passive coating, causing corrosion processes to begin.^1^ Reinforcement corrosion can lead to a variety of damages, such as the rebar's (steel reinforcement) loss in strength and ductility,^2^ and due to volumetric expansion of corrosion products, cracking in concrete cover,^3^ and bond deterioration at the steel–concrete interface as a result of cover confinement loss.^4^ Rust accrual causes tensile stress along the circumference of the steel rebar in the concrete cover because the amount of corrosion products is around two to six times that of steel. Numerous experimental and numerical studies have revealed that, under general corrosion, concrete cracking can occur at corrosion depths as little as a few tens of micrometers.^5^ Steel corrosion in reinforced concrete (RC) structures not only reduces the bond capacity but also alters its behavior based on factors like bond-slip characteristics in the uncorroded state. This shift is determined by whether the rebar is smooth or ribbed, whether transverse reinforcement is present, and whether splitting cracks form during pull-out before corrosion. The spalling or cracking of the concrete cover because of corrosion isn't just a structural durability issue—it directly affects the mechanical performance of the corroded steel. Pitting corrosion, which causes deep, localized damage, drastically reduces the strain capacity of the rebar. As a result, the failure mode can shift from ductile to brittle. Additionally, the load-carrying capacity of the corroded rebar is significantly compromised, directly correlating with the extent of cross-sectional area loss.^6^ These negative consequences all shorten the RC structures' service life and compromise residual safety. Over the past few decades, numerous empirical and theoretical models have been developed to assess the residual performance of corroded structures. These studies have identified several correlations between the degree of corrosion and essential performance metrics, such as the width of corrosion-induced cracks, bond-slip behaviour, mechanical properties of corroded reinforcement bars, and the load-bearing capacity of impacted structural elements. These insights stem from extensive research aimed at better understanding how corrosion impacts structural integrity over time. In contrast to laboratory specimens, it is challenging to precisely estimate the corrosion level for in-service structures or on-site before implementing those correlations for structural assessment.

Researchers have devised several detection techniques to measure the degree of corrosion on steel rebar in concrete. Various quantities are determined to indicate the corrosion state using different methodologies. For instance, the polarization resistance technique is typically used to measure the instantaneous corrosion rate. On the other hand, physical techniques like electromagnetic and electrical resistance measurements, 3D laser scanning, and X-ray micro-computed tomography can be used to characterize the accumulated degree of corrosion. Additionally, indirect methods are sometimes used to assess the corrosion state by analyzing factors influencing the electrochemical process of steel corrosion, such as chloride ion concentration, concrete pore solution pH, carbonation depth, and concrete resistivity. Moreover, techniques like acoustic emission and optical fiber sensing are employed to monitor corrosion-related damage in reinforced concrete, helping to estimate the cumulative degree of corrosion at the time of measurement. Owners must first accurately analyze the corrosion of their existing RC structures before acting appropriately to maintain or repair them. Overvaluing the remaining performance of corroded structures would raise the risk to public safety, while an overly conservative appraisal of the structures would lead to needless replacements or demolitions. To assess the effectiveness of restoration or repair efforts for structures impacted by corrosion, corrosion detection is also essential. As a result, it is imperative to create precise corrosion detection techniques that work with current structures.

Considering the aforementioned, the primary aim of this review is to provide an overview of the state-of-the-art corrosion detection techniques for reinforced concrete, both in the laboratory and in the field. The evaluated methodologies' working principles, data interpretation, and current advancements are provided. The study's second goal is to illustrate the limitations and challenges associated with assessing the on-site detection of corrosion caused by chloride using these lab methodologies (Fig. 4).

### Corrosion in reinforced concrete

**Fig. 1:**
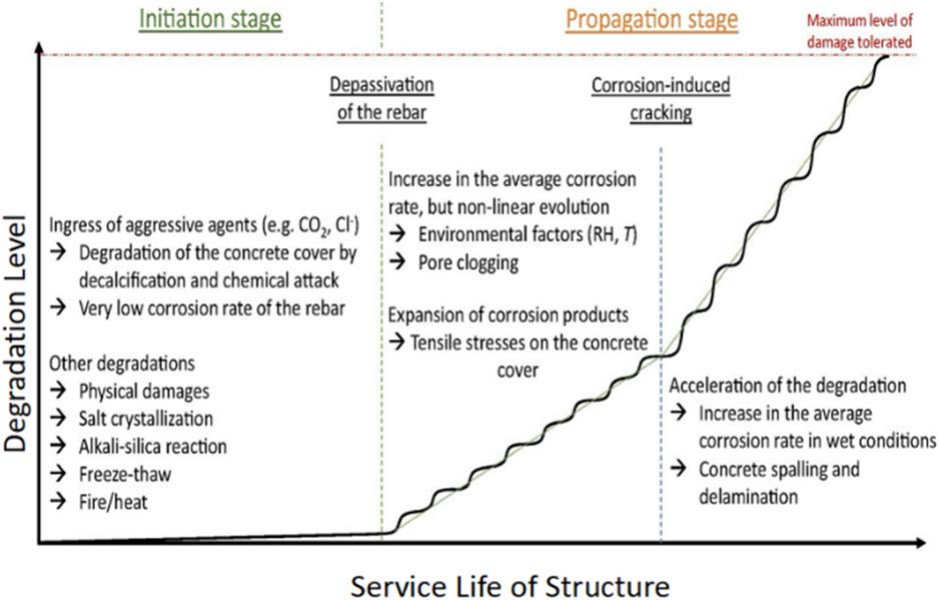
Schematic representation of the corrosion progression in RC, based on the Tuutti model, with consideration for the service life.^7,9,12^

There are essentially two phases to the lifespan of reinforced concrete structures, as proposed by Tuutti.^7^ The first stage is known as the initiation stage, during which aggressive agents such as CO_2_ and chloride penetrate the concrete and reach the rebar. Over time, the protective layer on the steel surface is deteriorated by these agents gradually, leading to corrosion and damage. The second stage, known as the propagation period, begins when the corrosion reaches a critical level and starts to spread rapidly, causing significant structural damage.^7^ Corrosion will remain in its active, propagating stage until it reaches the maximum allowable level according to building codes. Corrosion of steel is in its passive condition when the current density is either greater than 1 μA cm^−2^ or less than 0.1 μA cm^−2^.^8^ To distinguish between rust expansion, cover cracking, and spalling/delamination, subsequent models built upon Tuutti's model either took the corrosion rate variations over the reinforced concrete's lifetime into account or included extra steps in the service life determination process (Fig. 1).^9^ Although corrosion rates can increase due to cracking and spalling in concrete, they can also decrease when corrosion products fill the pores of the cracked concrete. The degree of degradation, therefore, is not linear.^10^ To improve the precision of service life prediction, models that incorporate electrochemical and transport processes are crucial.^11^

### Corrosion mechanism

The electrochemical process of reduction of oxygen into hydroxyl ion (OH^−^) at the steel surface, acting as the cathode, and oxidation of iron to ferrous ions (Fe_2_^+^) or ferric ions (Fe_3_^+^) depending on the environmental conditions at the anode, constituting the process by which steel corrodes in concrete.^12^ The primary anodic reaction implies the oxidation of iron to ferrous (Fe_2_^+^) and ferric ion (Fe_3_^+^).

1 Fe → Fe_2_^+^ + 2e^−^

In a highly oxidizing environment Fe_2_^+^ can be further oxidized to

2 Fe_2_^+^ → Fe_3_^+^ + e^−^

**Fig. 2:**
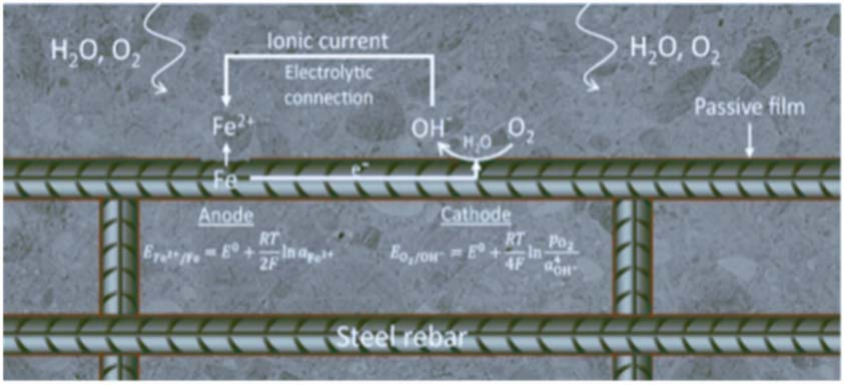
Corrosion process a schematic view of oxidation and anodic, cathodic reaction.^12^

One can also see the reduction of water/proton depending on the pH and available oxygen close to the surface of steel.^13^ Lastly, to transfer electrons, between the anode and cathode there must be an electrical connection, and an electrolytic environment is needed to transport ions in solution as shown in Fig. 2.^12^

Butler–Volmer^14^ equation provides the electrochemical kinetics of corrosion (eqn (1)):3
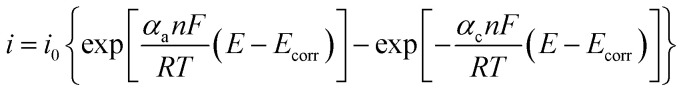
in the above equation, *R* and *F* represent the universal gas constant (8.31 J mol^−1^ K^−1^), and Faraday constant (96485 C mol^−1^), respectively. *E* is the electrode potential (V), *T* (K) is the absolute temperature, and *E*_corr_ is the corrosion potential (V). The variables in the equation are as follows: *i* represents the current density in (A m^−2^), *i*_0_ represents the corrosion current or exchange current density *I*_corr_ in (A m^−2^), *α*_a_ represents charge transfer coefficient of the anode, *α*_c_ represents the charge transfer coefficient for the cathode, and *n* represents the number of electrons transferred in the reaction. The expression 
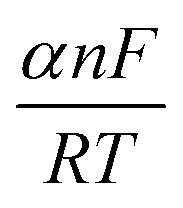
 is equivalent to 
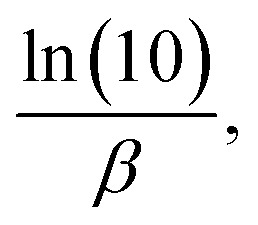
 where *β* represents the Tafel slope. In the presence of chloride ions, the anodic reaction (oxidation of iron) is enhanced, and the cathodic reaction (oxygen reduction) becomes more efficient due to the aggressive nature of chlorides, further accelerating the corrosion rate.

**Fig. 3:**
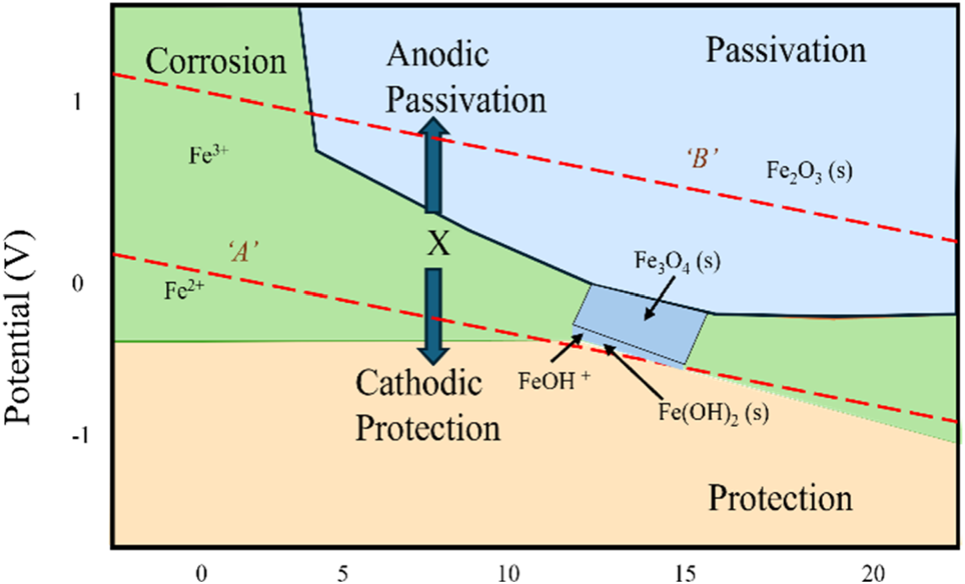
Pourbaix diagram for steel.^15^

Concrete often has a pH of 13 or above, making it alkaline. Hu *et al.*^15^ state that the main factor influencing whether steel is corrosive, or passive is pH. Steel reinforcement corrodes when two distinct materials react with the environment (such as oxygen, carbon dioxide, *etc.*) and produce oxides and hydroxides consequently. Moreover, it seems that a variety of chemical compounds in metals are synthesized in large part due to the electrochemical potential. The reinforcement's passive iron oxide layer is weakened by this reaction process, which also brings the pH down to less than 11. Among the many issues raised by this process is the weakening of the bond between concrete and steel, spalling and brittle failure. Fig. 3 illustrates the Pourbaix diagram, which sheds light on the concepts of immunity and passivation in the context of concrete reinforcement.^16,17^ Derived from the Nernst equation, this diagram plots the relationship between electrochemical equilibrium potential (*P*) and pH. The diagram categorizes the electric potential into three primary regions: (a) corrosion, (b) passivation, and (c) immunity. These classifications are based on the thermodynamic properties of pure metals. Additionally, the diagram includes two dotted lines, A and B, marking the boundaries for water electrolysis. Line A signifies hydrogen evolution, while line B represents oxygen evolution. The area below line B corresponds to the formation of hydrogen gas and hydroxide, whereas the region above line B relates to oxygen and proton evolution. In the passive zone, both Fe_2_O_3_ and Fe_3_O_4_ remain stable, helping the protective film resist corrosion. Fe_3_O_4_ also stands out for its high conductivity, which is attributed to electron hopping between Fe_2_^+^ and Fe_3_^+^ ions.

**Fig. 4:**
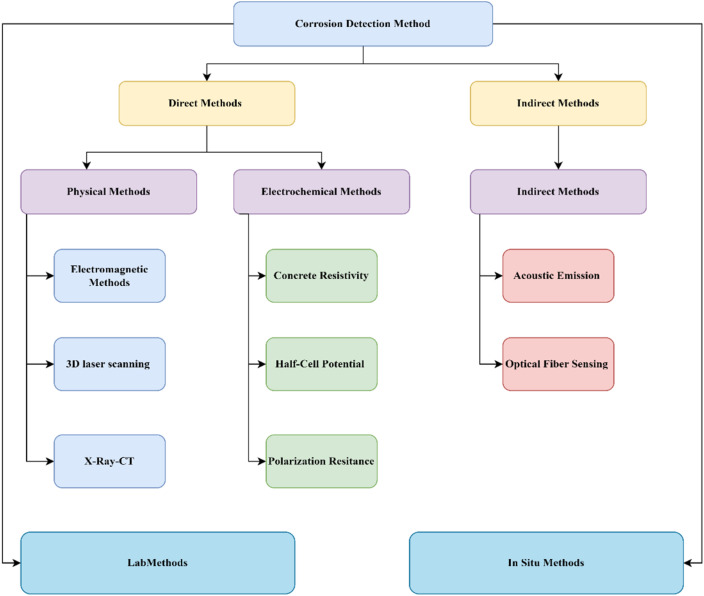
Flow chart for the chloride-induced corrosion detection method for the study.

## Chloride-induced corrosion (CIC)

### Chloride ion penetration in reinforced concrete

This section explains briefly about the chloride progression in RC. Chloride can infiltrate reinforced concrete through many pathways, such as construction water or materials that are polluted. It can also disperse from the surroundings, especially in marine regions with alternating wet and dry periods, or because of the use of de-icing salts in winter, such as CaCl_2_, MgCl_2_, and NaCl.^18^ Chloride mainly enters the body through capillary pores *via* capillary suction, diffusion, and penetration.^18^ The total chloride's diffusion coefficient in concrete, which represents the transport property, has a notable influence on the initiation time of corrosion. Precisely forecasting the extent of chloride penetration is difficult, as it can be influenced by a multitude of circumstances. The dissemination of concrete is affected by the distribution of pores and the connection between them, which are correlated with the water-to-cement ratio. To decrease the overall porosity, it is advisable to employ a low water-to-cement ratio (0.4–0.5) during the first stage extension. In addition, an elevated concentration of chloride ions can lead to chemical reactions with other substances, such as tri-calcium aluminate (C_3_A), resulting in the formation of Friedel's salt. Alternatively, these chloride ions may also adhere to other hydrates, such as C–S–H and monosulfoaluminates (AFm), by physical adsorption.^19^ The cement paste's specific surface area is mostly responsible for physical adsorption, whereas the paste's monocarboaluminate component is primarily responsible for chemical adsorption through Friedel's salt production.^20^ High calcium and alumina content SCMs may also affect the resilience of RC by reducing the ingression of Cl^−^ to the rebar and influencing the chloride binding capacity.^20^ Finally, Cl-diffusion is affected by temperature, water content, and electrical double-layer characteristics.^21^ In ITZs of concrete, the diffusion coefficient varies according to the volume and tortuosity of the material.^22^ The presence of CO_2_ and other harmful substances may lead to chloride entering the surface of the steel, which can be facilitated by cracks in the concrete or faults at the steel–concrete interface. It is important to figure out the rate of chloride ingression to model the service life of the initial stage of corrosion, irrespective of the parameters that affect the penetration of chloride.^23^

### Mechanism of chloride induced corrosion

When exposed to a sea environment or deicing salt, reinforced concrete structures frequently experience corrosion caused by chloride. Eqn (4) illustrates the function of chloride ions in the anodic process. Eqn (5) illustrates the cathodic reaction. The corrosion reaction requires chloride ions, which serve as both a catalyst and a carrier for the byproduct. However, they are not consumed or directly engaged in the reaction, as indicated by these equations.

4 Fe_2_^+^ + 2Cl^−^ + 4H_2_O → FeCl_2_·4H_2_O

5 FeCl_2_ + 2H_2_O → Fe(OH_2_) + 2HCl

Concrete can include either “unbound” or “chemically bound” types of chlorides. The general opinion is that corrosion in concrete is primarily caused by the existence of free chloride ions in the pore solution. Disregard bound chlorides as they are harmless. As a result of this comprehension, there are now strict limits on the permissible levels of chloride concentrations in concrete mixes. The maximum allowable chloride concentration, as specified by ACI 318, for concrete buildings that are exposed to chlorides during use is less than 0.15% of the cement's mass. On the other hand, the Chinese regulation GB 50666 specifies that the maximum allowable chloride content in cement should not exceed 0.06% by mass. According to British level BS 5328-1,^24^ the allowed range for the overall percentage of chloride ions in cement is between 0.1% to 0.4% by mass, depending on the specific quality level that the cement meets. When reinforced concrete structures are subjected to prestressing, their value is substantially reduced. Irrespective of the precise kind of cement employed, studies^25^ have indicated that bound chlorides can be just as detrimental. This is due to their capacity to undergo conversion into free chlorides, which can subsequently be released into the pore solution within a certain range of pH values (12.4–12.6). To mitigate the likelihood of corrosion, several codes such as ACI 222R^26^ have set limits on the chloride concentration in total, which includes both bound and free. In addition to the chlorides present in the basic components of concrete, environmental penetration can also lead to an increased presence of chlorides in concrete. Concrete's chloride diffusion coefficient and the thickness of the concrete cover control this process. Controlling the quality of the concrete (including the amount of chloride in the raw materials and the design of the concrete mix) and making sure that the concrete cover is thick enough can help to delay the occurrence of corrosion caused by chloride.

By pitting, chloride ions are the source of corrosion. Because of the way pitting occurs, corrosion caused by chloride is often limited in scope. The pH of the concrete pore solution is not influenced by the concentration of free chlorine, unlike carbonation-induced corrosion, which does modify the pH values.^27^ Despite a high pH level, the presence of free chloride ions can nevertheless lead to the de-passivation of steel due to the equilibrium between two conflicting processes occurring on the surface of the metal. The initial step involves the stability and repair of the film through the mediation of OH^−^ ions, whereas the subsequent process involves the breakdown of the film facilitated by free chloride ions. To promote the consistent expansion of pits following the removal of the protective layer, it is important to induce a localized decrease in pH. Repassivation will occur if the ratio of chloride ions (Cl^−^) to hydroxide ions (OH^−^) drops below a certain threshold. Thus, the ratio of chloride ions (Cl^−^) to hydroxide ions (OH^−^) has been proposed as a benchmark for the initiation of corrosion, as indicated by ref. 28. Upon crossing this threshold, the iron-oxide coating undergoes either permeability or instability, so rendering the steel no longer considered to be safeguarded against corrosion. The most suggested threshold for the ratio of chloride ions (Cl^−^) to hydroxide ions (OH^−^) is 0.59, as suggested by Gouda^29^ and Hausmann, even though the barrier appears to vary. Alonso *et al.*^28^ discovered that the threshold for mortars ranged from 1.17 to 3.98, whereas for synthetic pore solutions, it ranged from 0.25 to 0.8. Li and Sagues^30^ found that the threshold of [Cl^−^]/[OH^−^], which is pH-dependent, was elevated. The variability of chlorides' solubility, which is influenced by pH, leads to uncertainties regarding the applicability of the chloride (free state) concentration in the two parameters [Cl^−^]/[OH^−^] index. Several researchers suggest replacing free chloride ions in the [Cl^−^]/[OH^−^] two-parameter index with the total chloride concentration.^31^

## Corrosion detection methods

The introduction indicates that the corrosion process caused by chloride ions is influenced by several elements, including the penetration rate, water-to-cement ratio, and others. For determining the remaining service life of RC structures that have experienced corrosion, it is essential to accurately identify the conditions that led to corrosion, including the rate and quantity of corrosion, implementing effective strengthening and repair processes, and selecting suitable anti-corrosion treatments. This review, based on the literature available for chloride-induced corrosion detection is categorically divided into two *i.e.* lab and *in situ* investigation, which is further divided into direct and indirect methods. The flow chart below shows the details of this process of review.

The presence of CIC in reinforced concrete buildings may be detected using a variety of approaches. Depending on the availability and resources, lab investigation can be done in direct or indirect methods which are further classified based on their procedure into different categories. The section below will discuss in detail each of the methods.

## Direct method

The corrosion parameters, including corrosion rate, residual steel content (the residual steel content means the amount of steel left after corrosion may picture the better results in determining the corrosion level as shown in Fig. 6(a)), and corrosion rate are monitored by direct techniques. Direct detection techniques are described as physical and electrochemical techniques, respectively, by the testing principles.

### Physical methods

Physical methods for detecting chloride-induced corrosion in RC structures often rely on non-destructive testing (NDT). Techniques like electromagnetic sensors or ground-penetrating radar assess chloride infiltration and pinpoint areas susceptible to corrosion. Visual inspections for surface rust on reinforcing bars also provide a valuable initial assessment of structural health and potential repair needs.

### Electromagnetic method

**Fig. 5:**
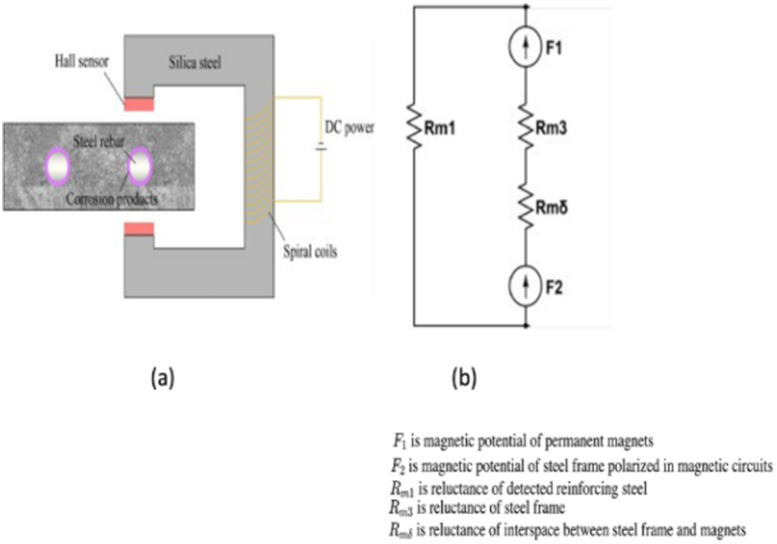
(a) Electromagnetic corrosion detection device schematic diagram, (b) equivalent model for the electromagnetic set up.^34,35^

Various devices based on the principle of electromagnetic (EM) have been established to detect RC corrosion. The operating concept is that various materials have varied magnetic properties: iron oxides, concrete, and air have magnetic permeabilities that are about a hundred times lower than those of carbon steel.^32^Fig. 5 depicts the operating principle schematically. The observed RC specimen experiences a drop in the cross-sectional area of steel and a shift in magnetic flux lines corresponding to the formation of corrosion products when subjected to an electromagnet-generated magnetic field. The strength of the magnetic field is detected and measured using Hall-effect sensors.^33^ Applying the same concept, EM non-destructive technology is also used to find steel rebar in concrete and measure the amount of water in fresh concrete.

Researchers have lately created a variety of high-tech electromagnetic sensors to track the corrosion of steel in concrete. Their use allowed for the localization of corrosion zones and the linking of magnetic response to steel mass loss. Rebar used at higher depths within a concrete structure is more sensitive to electromagnetic sensors. The success of the electromagnetic method may be disturbed by how the corrosion thickness is aligned with the magnetic flux, albeit this is an important consideration. If the direction of the applied magnetic field and the thickness of the corrosion are the same, then a layer of corrosion product can block the flow of magnetic flux. Therefore, changes in magnetic intensity are becoming more and more significant.^36^

### 3D laser scanning

**Fig. 6:**
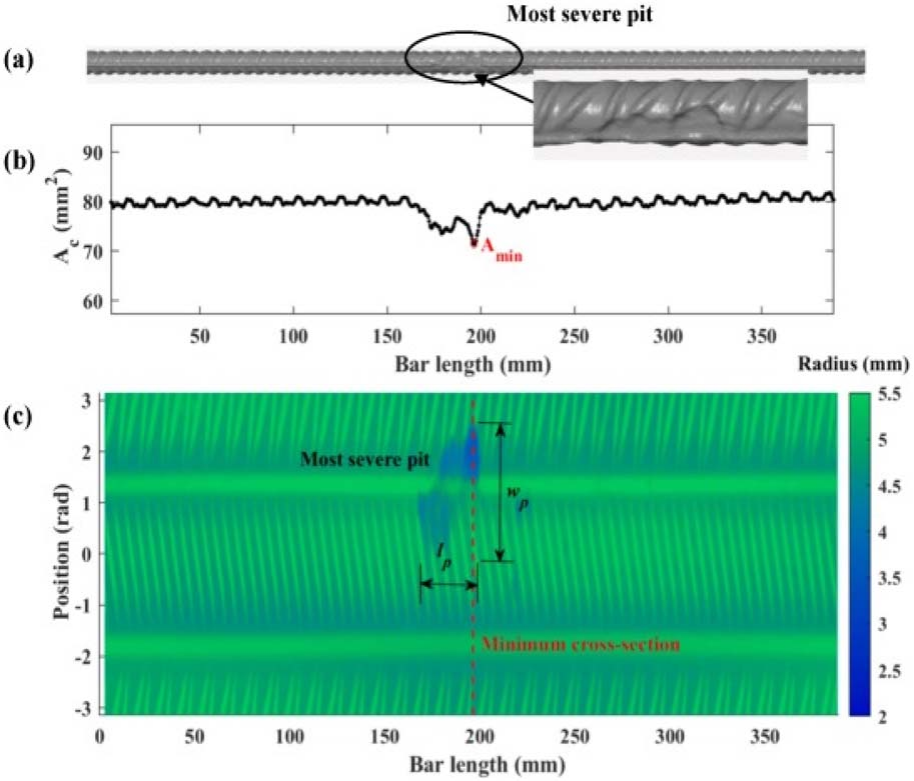
Example of the result of 3D scanning of an experimental sample (a) rebar reconstructed surface, (b) change in cross sectional area of rebar across the length, (c) a colored 2D plot for the radius of rebar.^39^

Three-dimensional (3D) laser scanning is a visual technique that uses light pulses to generate a three-dimensional representation of an object's surface. It is extensively utilized in the field of industrial engineering. To facilitate the scanner's identification of surface locations as reference points, it is necessary to affix small adhesive targets onto the test object. Recently, this method has been used to evaluate rebar corrosion, considering their shape and quantity.^37^ By analyzing 3D scanning data, it can accurately determine the change in cross-sectional area, distribution of pit depths, and the overall volume or mass loss of steel in rebar. This enables the establishment of the “corrosion level” by considering other criteria such as the depth of pits, the geometry of pitting, and the probability distribution of the loss of cross-sectional area. Fig. 6 illustrates the process of assessing corrosion by utilizing 3D scanning of degraded reinforcement bars. The 3D laser scanning method allows for the analysis and measurement of the reduction in cross-sectional area, as well as the identification of localized pits in terms of their length, width, depth, and distribution along the reinforcement bar. This data may be utilized to establish a more accurate correlation between the extent of corrosion and the mechanical properties of corroded steel rebars. Furthermore, the corroded rebar may be 3D scanned to create a digital model. This model can then be used to construct a geometric model in finite element modeling. This allows for the prediction of mechanical characteristics of reinforced concrete buildings and corroded rebars.^38^

### On-site measurement

Regrettably, the process of using 3D laser scanning to detect corrosion requires the removal of rebars from the concrete. This procedure is destructive and thus makes it impossible to monitor the corrosion status of the same RC sample at different time intervals. This is the fundamental reason why it can't be used in reinforced concrete structures that are constructed on-site.

### X-ray micro-computed tomography

**Fig. 7:**
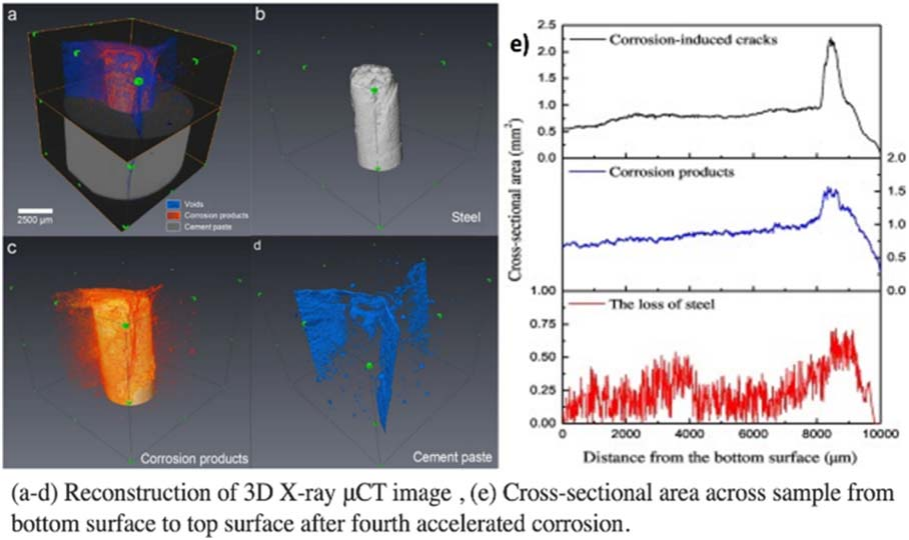
X-ray micro computed tomography results.^42^

The popular non-destructive imaging approach known as X-ray micro-computed tomography (X-ray μCT) was initially created for medical applications. It enables a three-dimensional examination of a sample's interior structure. In cementitious materials, it has recently been used to inspect the processes of corrosion in steel.^40,41^ The foundation of the X-ray μCT technology lies in the fact that various materials possess varying capacities for X-ray adsorption. Based on the makeup of the penetrating materials, the X-ray beam's intensity attenuates to differing degrees when it passes through an RC sample as detected by a detector. Corrosion products, steel rebar, concrete flaws (pores, voids, and fractures), and cementitious components, may all be recognized and discriminated against, according to research.^42^ For instance, Fig. 7(a)–(d) shows a rebuilt 3D tomography of a reinforced concrete sample that was exposed to corrosion. By extracting distinct objects, the authors were able to quantify the cross-sectional areas of various targets, such as corrosion products, cracks, and steel loss, along the sample's height, as shown in Fig. 7(e).^42^ Rossi *et al.*^43^ conducted a study for the evaluation of 20 years old chloride-induced specimens by X-ray CT method. The investigation demonstrated that the sites where corrosion had gotten the deepest were correlated with interfacial voids. Image analysis revealed the presence of nearby corrosion pits at every place at the SCI where there was an air vacuum. Interfacial voids should be regarded as deleterious corrosion sites if the concrete is partially or completely wet and there is a specific chloride concentration at the SCI.

### On-site measurement

With X-ray CT, both qualitative and quantitative *in situ* methods may be used to track the progression of steel corrosion in concrete over time. It is possible to determine the corrosion distribution, cumulative corrosion quantity, and temporal variation of corroded steel reinforcements using 3D photos. Other methods of corrosion detection struggle to do this. Because of its capabilities, X-ray CT is a perfect instrument for studying the mechanisms behind corrosion build-up, internal damage caused by corrosion to concrete, and rust penetration into pores and fractures in labs. Nonetheless, the X-ray μCT technique's great resolution necessitates that the examined samples be tiny, often measuring less than a few hundred millimeters. Furthermore, the limited applicability of existing X-ray μCT devices is due to their expensive prices and specific criteria. Small samples must be taken from the site and sent to labs to evaluate the existing structures.

### Electrochemical methods

Conventional electrochemical devices can reflect the electrochemical response that is the corrosion process. Steel corrosion potential in concrete may be measured using one of three classic electrochemical techniques: the electrochemical impedance spectrum (EIS) method, the half-cell potential method, or the polarization curve method.^44^ The electrochemical impedance properties of mortar in sulfate and chloride conditions were investigated by Bragança *et al.*^45^

Their findings indicated that the mortar had capacitive characteristics at lower frequencies when exposed to the sulfate environment. The corrosion behavior of rebars in an open-air atmosphere was evaluated by Thee *et al.*^46^ using electrochemical impedance spectroscopy (EIS). A mathematical analysis approach was also suggested to determine the loss of mass in rebar corrosion directly by EIS measurement. Electrochemical devices and wireless signal transmission techniques were integrated by Qiao *et al.*,^47^ who used micro-energy produced by steel corrosion as the primary monitoring index. Caines *et al.*^48^ developed the electrochemical noise method (EPN) to explain the correlation between mass loss and reinforcement corrosion rate, grounded in the core principles of electrochemical theory. To emphasize the impact of rust inhibitors, Morris *et al.*^49^ considered several electrochemical technology indices, including electrochemical impedance spectrum, corrosion voltage, and corrosion current. They also computed the critical chloride concentration, which is impacted by the water/binder ratio.

Using acoustic emission technology and an electrochemical noise approach, Dhouibi *et al.*^50^ assessed the corrosion state of stainless steel 304. Compared to pitting corrosion and its subsequent shift to uniform corrosion, stress corrosion is empirically proven to occur more often. Karthick *et al.*^51^ developed a method to monitor the corrosion of steel bars within concrete by implanting an electrode in the concrete. This approach holds great promise for engineering applications. However, the electrochemical system is vulnerable to damage from reinforcement, and its complex concept and the requirement for the test specimen to be immersed in a solution make it difficult to implement in real-world engineering scenarios. Nonetheless, corrosion potential is an important measure to judge the corrosion state of reinforcement according to electrochemical theory.

### Half-cell potential (HCP)

Hall cell potential or corrosion potential is the open circuit potential of rebar. In a two-electrode setup for measuring the electrical potential difference, a high-impedance voltmeter is utilized to connect the reference electrode (RE) which represents one half of the cell to the rebar, which represents the other half of the cell. The figure below illustrates this arrangement.^52^ The generally recognized procedure for determining the half-cell potentials of uncoated reinforcing steel in concrete is the ASTM C876 (ref. 53) standard test technique. It is typically necessary to chip away at the concrete cover to make sure the rebar is properly engaged, as it is not easily accessible.^54^ The reference electrodes consist of saturated camel electrodes (SCE), copper sulfate electrodes (CSE), and silver chloride electrodes. Most of these electrodes are typically marketed as gel- or liquid-filled electrodes. To be fixed on the surface of the concrete, this form of RE requires effective electrolytic contact. To minimize junction potentials as much as feasible, this is often guaranteed using a wet sponge with a suitable solution that has a pH like a pore solution.^55^ All studies must disclose this information since the kind of electrolytic contact and the electrode location have an impact on the OCP readings.

Electrodes made of graphene and cement, as well as those embedded in concrete and made of solid-state metals or metal oxides (MnO_2_, activated carbon, graphite, *etc.*), are stable over months or even years.^56^ Another affordable sensing technology is offered by screen-printed electrodes based on silver. This is especially intriguing for newly constructed buildings as the electrodes may be inserted immediately during the building process, but they can also be applied to already existing buildings during maintenance procedures.^57^ Since the pore solution guarantees electrolytic contact with embeddable electrodes, improving the data quality, it is anticipated that the liquid junction potential will remain more stable with time. The issue of contact resistance is also less troublesome. However, because the electrodes are fixed, the system has less flexibility.^34^

### On-site measurement

Several researchers have attempted to develop a quantitative relationship between the corrosion rate and the corrosion potential, but none have proven successful. For tiny specimens, there is a strong association between the two parameters when measurements are done under extremely controlled circumstances (RH, *T*), particularly in the high corrosion probability band.^58^ The environmental conditions in field research, however, are not as controllable. Various factors can impact measurements, such as the state of the steel rebar (the proportion between the cathode and anode), the proximity of oxygen to the steel surface, the makeup of the pore solution (including sulphide content, pH level, and chloride content), as well as the depth, shape, resistivity, and presence of cracks in the concrete cover.^59^ It is important to emphasize that the half-cell potential measurement only provides the likelihood of corrosion at a certain location and time. It makes more sense to track the half-cell potential data over an extended period.^60^ The method most frequently used to assess the corrosion of steel in concrete is HCP measurement. To accurately assess the corrosion of reinforcing bars used in construction, it is recommended that measurements begin no earlier than 15 minutes after the initial measurement's wet area. Additionally, it's suggested that a substantial portion of the building be completely saturated. Except for fundamental research on the corrosion processes, the reinforcing bar and its surface condition should be typical of those used in practice to acquire more useful findings.^61^ In concrete with low resistivity, the potential distribution across the concrete surface closely mirrors that at the steel/concrete interface. However, as the concrete's resistivity increases, the surface potential distribution can significantly deviate from that at the steel/concrete interface. This discrepancy is most noticeable near the transition zone between the anode and cathode.^62^

HCP readings can be misleading without considering concrete resistivity. The same corrosion rate can cause different surface potentials due to varying resistivity. This leads to multiple potential readings for the same corrosion state. As concrete cover thickness increases, surface potential diverges from the steel–concrete interface, making surface readings inaccurate. While HCP works with or without oxygen, its impact is minimal in oxygen-free environments.^62^

### Concrete resistivity

**Fig. 8:**
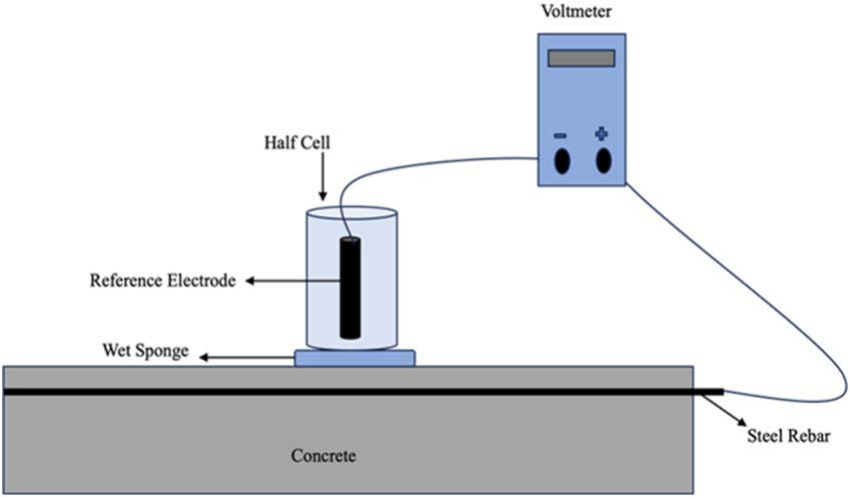
A schematic diagram for the experimental setup of half-cell.

Electrical resistivity, or concrete resistivity (*ρ*, measured in Ω m), is a material's capacity to resist electrical circulation.^63^ Concrete typically has an initial resistivity of 10–106 Ω m.^64^ The water-cement ratio, size of aggregates, type of binder, and curing and storage conditions all affect the strength of concrete because they change the composition of the pore solution and the porosity of the concrete.^65^ These factors are crucial because they control the corrosion process. For the duration of RC structures' service lives, it is anticipated that the resistivity would fluctuate due to the deterioration of the concrete triggered by the entry of hostile chemicals or the development of corrosion-induced fractures. Consequently, monitoring concrete resistivity is essential for assessing the evolution of concrete durability and the corrosion process (Fig. 8).

According to eqn (6), the resistivity and resistance of concrete are directly correlated *R*_Ω_ (Ω).6
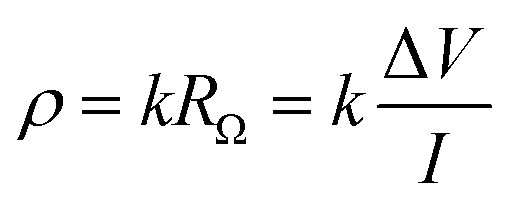


**Fig. 9:**
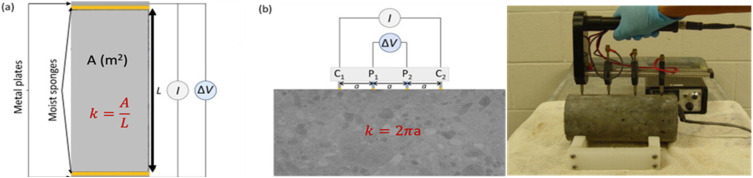
A schematic diagram of resistivity measurement (a) uniaxial configuration for bulk resistivity, (b) Wenner configuration schematic diagram (left), experimental setup for Wenner configuration (right).^68,69^

Various approaches may be employed to determine the resistivity of concrete. It is dependent upon a geometric factor (*k* in m), the potential difference (Δ*V* in V), and the injected current (*I* in A). This component takes into account the dimensions and shape of the sample, as well as the experimental equipment employed.^64^ The bulk resistivity cell, designed in a uniaxial manner as shown in Fig. 9(a), employs two metal plates arranged in parallel with wet sponges at both ends of the concrete specimen. A potential difference between the two plates is measured after an electric current is applied to them to get the resistivity.^66^ The geometric factor *k* in this instance is:7
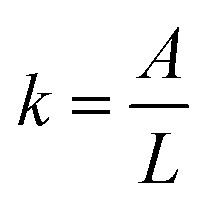
where *L* and *A* are the length of the sample (m), and the perpendicular cross-sectional area (m^2^) of the current respectively.

Despite being quick to measure, resistivity is mostly measured in lab settings and is rarely useful in outdoor operations. Concrete resistivity may be measured using a device with a four-electrode assembly, usually using stainless-steel probes. Two electrodes, labeled as C1 and C2, are responsible for injecting an electric current, while another two electrodes, labeled as P1 and P2, are used to measure and detect potential differences. Not only are the linear and square-array four-point probes popular, but there are also additional designs that may be used, such those with embedded probes.^67^ The geometric factor for the Wenner design (Fig. 9(b)) is determined by considering the two internal electrodes, P1 and P2, which have a similar probe spacing of *a*(*m*), as well as the two external electrodes, C1 and C2.8
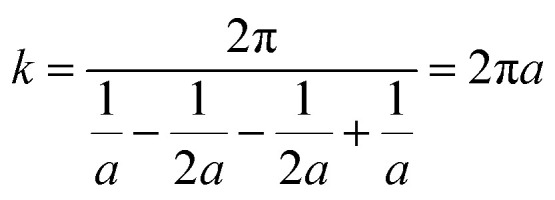


It is crucial to acknowledge that when applying this relationship to small concrete samples, it is assumed that the concrete is uniform in composition, exhibits the same properties in all directions, and has an infinite extent in one direction. To achieve precise calibration of the device, it is possible to utilize an electrolyte with resistivities that are already known. This electrolyte may be placed in a core holder that has a comparable shape to the concrete sample. Alternatively, numerical simulations can be employed to ascertain the accurate geometric factor and rectify boundary effects.^70^ The measurements may also be impacted by the kind, size, and method of the electrodes' electrolytic contact with the concrete surface, as well as by the frequency of the measurements. To prevent electrode polarization, measurements of concrete resistivity that may be performed in both DC and AC modes are often performed in AC mode.^71^ In general, measurements are performed in the bulk configuration's frequency (0.5–10 kHz) and the Wenner configuration's frequency ranges 0.01–10 kHz.^72^ To get an accurate, read on the concrete's strength, impedance spectroscopy is required to find the frequency when the reactance, a part of the impedance, is at its minimum. Since this frequency changes with the concrete's moisture level and microstructure, it must be determined case-by-case.

By using the modified parallel law (eqn (9)) and considering the concrete resistivity, it is possible to get the formation resistivity factor (*F*_R_). The *F*_R_ characterizes the microstructural properties of the concrete:^73^9
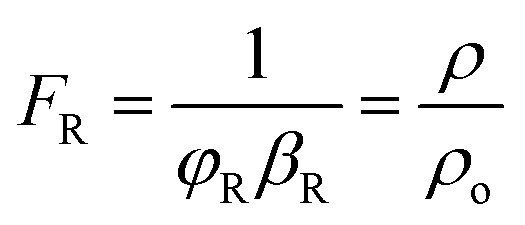


For a bulk sample, the resistivity is represented by *ρ*, whereas the resistivity of the pore solution is denoted by *ρ*_o_. The porosity and connectedness of the system are represented by the symbol *φ* sub cap *R*.^74^ The ratio of the pore resistivity of the solution to the bulk resistivity of the sample is another definition of the formation factor. Thus, it may be applied to ascertain the pore tortuosity and capillary porosity of both fresh and hardened concrete. The diffusion coefficient and this component are also connected by the Nernst–Einstein connection eqn (10) below:^75^10
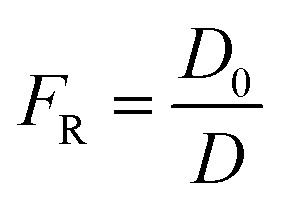
where *D* and *D*_0_ are the effective diffusion and self-diffusion coefficients (m^2^ s^−1^) of the ionic species in water. Fick's 2^nd^ rule of diffusion^76^ can be used to calculate the diffusion coefficient of chloride in concrete, allowing for the prediction of the time to corrosion once either experimentally or conceptually, the pore solution's resistivity has been measured.^77^ The formation factor can also be used with the Freundlich or Langmuir adsorption isotherm, and the Nernst–Planck equation^78^ or the concrete's coefficient of apparent chloride diffusion^79^ can be used to forecast chloride ingress.

Numerous factors that might negatively impact the formation factor calculation are impacted by the concrete resistivity measurement. The two primary parameters are temperature and water content: an increase in either of these triggers a reduction in the resistivity of the concrete and an increase in ionic transport in the pore solution.^64,80^

In summary, a comprehensive correlation between the corrosion rate and the resistivity of concrete could not be established.^81^ Research has shown that both the resistivity of concrete and the resistance to polarization are influenced by changes in temperature. The former phenomenon may be represented using an equation of the Arrhenius type,^82^ whereas the latter can be described by the Eyring law. This distinction may provide insight into the absence of a universal connection between the two. In summary, after identifying the kind of cement, it may be beneficial to utilize concrete resistivity as a means of assessing the non-destructive potential for rebar corrosion and approximating the extent of the corrosion rate. Like the half-cell potential approach, the resistivity gradient may also be utilized to assess the likelihood of corrosion and provide valuable insights. Nevertheless, it is not suitable as a conclusive method for correctly calculating the corrosion rate.

### On-site measurement

**Fig. 10:**
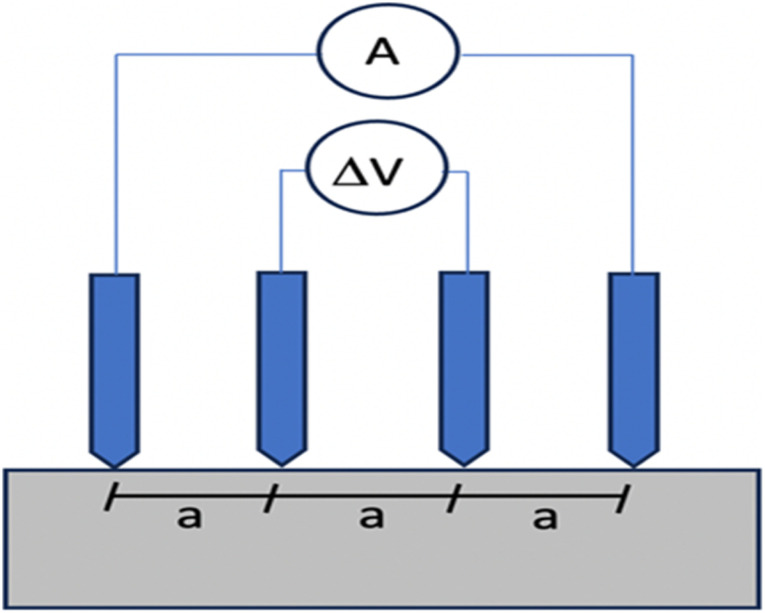
Concrete resistivity measurement by four electrodes.^83^

The RILEM^83^ recommendations recommend the use of the Wenner or 4-point approach for conducting on-site measurements of concrete resistivity. This technique entails utilizing a probe equipped with four-point electrodes that are equally distributed on the surface of concrete. This enables the measurement of resistivity without the necessity of implanted electrodes. Fig. 10 demonstrates that the two inner electrodes are responsible for measuring the potential drop, whereas the two outside electrodes are responsible for generating current. Resistance is defined as the quotient of the electric current divided by the potential difference. The volume of concrete through which current flows is approximately proportional to the distance between the electrodes. A conductive liquid is put into the concrete to enhance the contact between the electrode points. Contact resistance between the electrodes and the concrete, among other factors, has been shown to cause measurement errors in some cases.^84^ Two other alternatives include pressing two electrodes into the surface or using the reinforcement as the second electrode and placing one electrode on the surface.

For optimal results in most investigations, it is advisable to utilize sinusoidal AC with a frequency ranging from 50 Hz to 1000 Hz. The use of direct current (DC) may lead to inaccuracies caused by electrode polarization. Throughout the experiment, the resistance value is assessed, which is contingent upon the electrode geometry and necessitates conversion to resistivity. Resistivity is an inherent characteristic of a material that remains unaffected by its dimensions or magnitude.

The fundamental idea behind the electrical resistance (ER) technique of corrosion detection is that a metal's resistivity is determined by its shape. Steel's electrical resistance increases as corrosion reduces its thickness. ER probes can determine cumulative steel mass loss by measuring electrical resistance changes. Assuming uniform corrosion, the average corrosion rate can be computed over specified periods. Since a metal's resistance is temperature-sensitive, *in situ* compensation must be provided by correcting the observed resistance values to remove the impact of temperature. This may be done by inserting an uncorroded ER probe in the same environment.^85^

Because severe localized corrosion may result in very little overall loss of metal and the resistance change of the sensor may remain unnoticeable, the ER approach is more accurate in detecting general corrosion than localized corrosion. The ER method's long reaction time is another disadvantage. It might take many weeks for corrosion damages to build up to a point where they can be recognized when the rate of corrosion is minimal.^86^ It may therefore be difficult to gauge when corrosion begins at a low corrosion rate. To overcome this issue, we may make use of recent advancements in optimizing electrical circuits and materials to make the ER probes more sensitive.^87^

Although there are now many uses for ER probes in petrochemical engineering, there aren't many in the cementitious materials industry. The viability of using ER techniques in concrete to calculate the corrosion depth and mass loss quantitatively was demonstrated by Živica.^88^ Steel wires were longitudinally inserted into masonry blocks by Cella and Taylor,^86^ who then used electrochemical methods including LPR, HCP, and EIS together with ER to assess the corrosion behavior. The outcomes demonstrated that the ER approach outperformed electrochemical tests in terms of corrosion rate sensitivity and definitiveness. ER probes are sensors that can only monitor specific areas and thus, a sensible configuration of installation sites must be considered before integrating them. Gartner *et al.*^89^ provided reliable corrosion monitoring data over 52 months. They achieved this by putting multiple ER probes in reinforced concrete columns that were exposed to different areas in an environment with marine conditions. To address the shortcomings of the ER method in localized corrosion, Legat *et al.*^90^ integrated ER and EPN (electrochemical noise) approaches to observe the corrosion of steel in RC during consecutive wet–dry cycles. As an outcome, their research revealed the feasibility of tracking both the specific and overall levels of corrosion. It is important to mention that in reinforced concrete constructions when ER probes are used, the sensing material on the probes is employed to quantify the amount of corrosion, rather than measuring the corrosion on the rebar itself. Although the ER probes are near the steel being investigated, they may encounter different corrosion conditions.

### Polarization resistance

Rebar resistance to oxidation when an external voltage is applied, or when the rebar is polarized, is known as polarization resistance (*R*_p_, expressed in Ω). Using three- or four-electrode setups, several electrochemical techniques have been devised for calculating *R*_p_.^91^ The various methods that will be covered in this section are predicated on polarizing the rebar by applying an external disturbance to the system. The electrochemical noise technique is currently underexplored in the context of RC structures, despite its potential as a valuable tool for corrosion research. This method allows for measurements to be completed without causing any electrical interference.

**Fig. 11:**
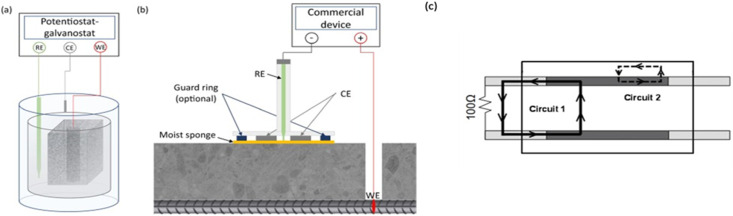
(a) schematic diagram for the lab testing by polarization resistance method usually a small size specimen, (b) field monitoring of corrosion by polarization resistance method generally more sophisticated equipment are used, (c) an equivalent different circuit diagram based on three-electrode system ASTM G109 device.^34^

The three-electrode configuration consists of a steel rebar as the working electrode (WE), a counter electrode (CE) made of titanium, platinum, or stainless steel to complete the electrical circuit, and a reference electrode (RE). Fig. 11 displays the schematic diagram. Given that each of the three electrodes can influence the measurement, it is imperative to provide a detailed account of the electrode configuration.^92^ Hence, all research investigations must incorporate details about the composition and structure of the CE, the nature and placement of the RE, and the choice between embedded or surface electrodes with a designated electrolytic connection.^93^

**Fig. 12:**
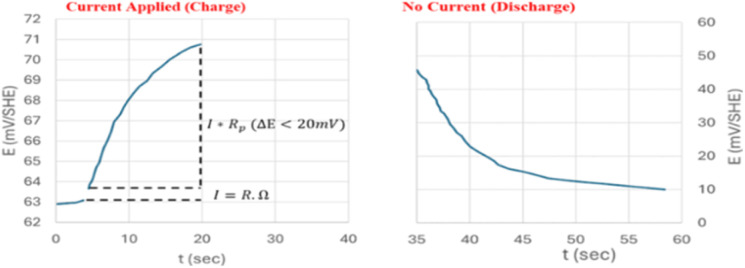
A graph illustrating the GP measurement of the anodic direction with a 50 μA injection over 30 s, revealing the change in Δ*E* over time throughout the charge and discharge phases.^34^

Using a modest potential sweep applied to the rebar around its open circuit potential (OCP) which in general is ±10–20 mV, either in the cathodic or anodic direction, the linear polarization resistance (LPR) approach measures the ensuing current an example of the measurement is shown in (Fig. 12). An alternative method of doing the measurement is to record the potential that results from applying a current sweep.^94^Eqn (11) provides the polarization resistance, which is the tangent of the potential–current curve at zero net current.11
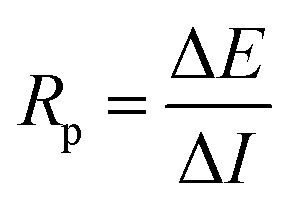


It should be noted that the slope is the product of concrete resistance and polarization resistance rather than being exactly equal to *R*_p_.^16^ To accurately calculate the values of *R*_p_, it is consequently required to adjust for the ohmic drop *R*_Ω_.^95^

**Fig. 13:**
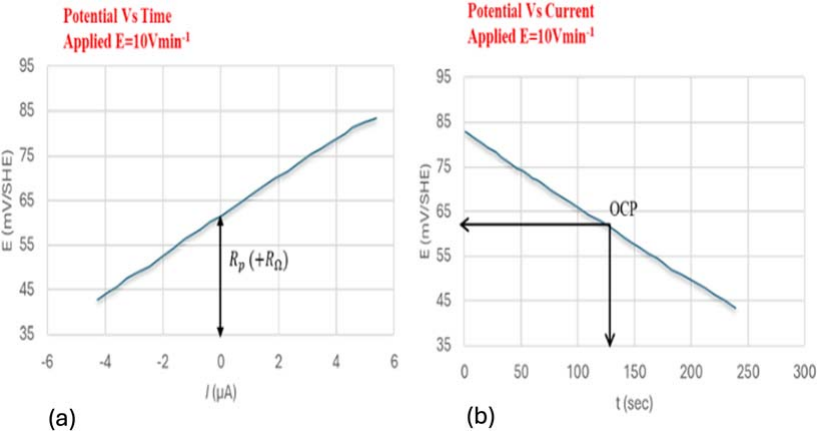
An illustration of an LPR measurement in relation to OCP in the cathodic direction, from ±20 mV. (a) Potential's evolution over time using a potential sweep of 10 mV min^−1^. (b) The potential's evolution with a current during the potential which makes it possible to calculate *R*_p_.^34^

The pulse technique is the second way that *R*_p_ is calculated. Even though they can also be performed in potentiostatic,^96^ or coulostatic mode,^97^ the measurements are often performed in galvanostatic mode.^98^ In the galvanostatic mode (also known as the galvanostatic pulse, or GP), a brief period (usually 5 s to 30 s for active corrosion) is spent applying a low anodic current (*I*_app_ = 5–500 μA) in either the anodic or cathodic direction to the reinforcement. The potential that occurs temporarily is recorded until it stabilizes.^95^ Concretes' ohmic resistance, *R*_Ω_, causes a substantial potential rise shortly after polarization. The electrical double-layer effect then causes a further gradual increase till it reaches a stable state (Fig. 13). The potential change should not be greater than 20 mV to validate the assumption of linearity between current and potential.^95^ When the current is cut off, the discharge exhibits behavior that is comparable to what was observed during the charging.

Various techniques have been devised to measure the polarization resistance on a concrete surface using a four-electrode array without direct contact with the rebar, commonly referred to as indirect polarization.^99^ The predominant Wenner configuration consists of four electrodes evenly spaced apart (as seen in Fig. 9).

A direct current^100^ or an alternating current^101^ applied at varying frequencies may be used to perform measurements. A geophysical technique that is similar to the AC approach is spectral-induced polarization (SIP), which is also known as indirect EIS. In contrast, the DC method is called indirect GP, which is analogous to TDIP in geophysics. Using polarization resistance as an approximation of the corrosion rate of steel CR, one may determine the corrosion current *I*_corr_. To calculate the corrosion current, one uses the Stern–Geary relation, as shown in eqn (12) below,^102^ which is in line with the RILEM standard.^95^12
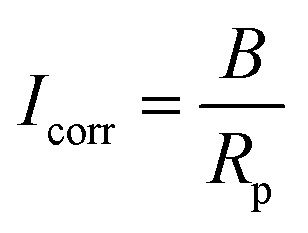
in which *B* represents the Stern–Geary constant (V). The commonly believed values of *B* for the passive and active corrosion of steel in concrete are 0.052 V and 0.026 V, respectively,^103^ which do not adequately capture the intricacy and temporal fluctuation of the corrosion process.^104^ According to eqn (13) precise values of *B* should be ascertained analytically:13
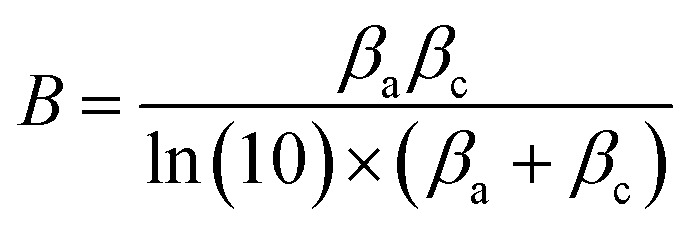


The Tafel constants can be calculated by applying the “Butler–Volmer equation” (eqn (1)). This equation states that the two coefficients can be obtained by plotting log(*I*) against the overpotential *η* (*E* − *E*_eq_). However, determining Tafel slopes is not always feasible in practical applications, as the anodic part of the polarization curve can be nonlinear, making it challenging to measure accurately.^105^ Furthermore, a significant disadvantage of this method is that the intense polarization that occurs during the Tafel scan may result in permanent alterations to the steel rebar.^106^ By utilizing the suggested values of “0.026 V and 0.052 V” for *β*_a_ and *β*_c_, respectively, which have been derived from experimental data and documented in scientific literature, it is still possible to accurately calculate the CR. In most cases, this is achieved with an error margin of less than one standard deviation.^107^

When studying uniform corrosion, Faraday's law is often used to convert the current density (*I*_corr_) to the corrosion rate (CR) as expressed in eqn (14). In this equation, *M* represents the molar mass of iron (in g mol^−1^), *F* represents the Faraday constant (in C mol^−1^), *M* is the mass loss (in g s^−1^), and *I*_corr_ is the corrosion current (in A). Eqn (15) provides the corrosion penetration rate (in cm s^−1^).14
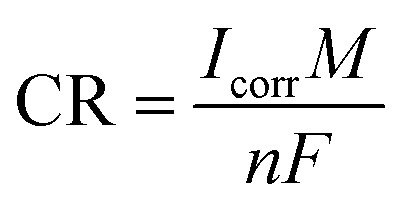
15
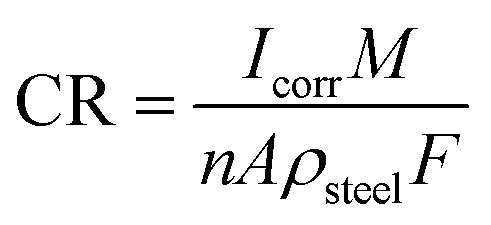
where *ρ*_steel_ represents the density of steel (g cm^−3^) and A represents the surface area of the rebar (cm^2^). A current density of 1.0 μA cm^−2^ corresponds to a corrosion rate of 11.59 μm per year, assuming that the corrosion of black carbon steel is uniform.^8^

To evaluate the outcomes obtained using the direct and destructive method, it is common practice to conduct gravimetric weight-loss tests as per standard guidelines (ASTM G1). These tests provide an indirect assessment of the polarization resistance. Given the known polarized area, it can be inferred that the LPR, GP, and EIS methods are capable of accurately predicting the corrosion rate of actively corroding rebars in the laboratory environment.^108^ To find the best method for every circumstance or to get an average, it's best to try out a few various methodologies. Variations in *R*_p_ values may be identified by taking characteristics like sweep rate, waiting time, polarization time, and applied frequencies into account.^109^

### On-site monitoring

Since the polarized area is typically unknown for field studies, the precision of these methods may be constrained. Through laboratory experiments, Fahim *et al.* showed that an appropriate EEC in the four-electrode configuration can accurately estimate the corrosion rate of highly resistive concrete, for both active and passive corrosion,^110^ confirming the configuration's great interest. To find out whether this method can be used to measure the rate of corrosion on reinforced concrete structures, more research is still needed.

It is important to remember that Wagner and Traud's mixed-potential theory, which was used to define the relationship provided by Stern–Geary (eqn (10)) for uniform corrosion, is fundamentally inapplicable to the corrosion of rebar in reinforced concrete structures, where macro cell corrosion is anticipated.^111^ For a similar reason, it is often not valid to utilize Faraday's rule to calculate the penetration rate, particularly when it comes to corrosion caused by chloride.^112^ To determine the localized corrosion rate using GP data, a novel theoretical technique has been put out.^113^ By using this method, we can lower the overestimation of the corrosion rate to a maximum of 2, which is much better than the traditional Stern–Geary method which can result in overestimation by a factor of 10 or more. The method considers two factors that affect the behavior of the macro cell elements when excited. The first factor is that the anodic element receives only a portion of the injected current, and the second factor is that this current varies during the measurement. Empirically, the cover depth, concrete resistivity, and steel surface area around the concrete surface may be used to estimate two parameters in engineering practice. This is achievable since these characteristics may be readily obtained in RC structures.^113^ If the analysis of GP results using the typical three-electrode setup has indicated a similar approach, it should also be applied to the four-electrode design. RILEM has provided guidelines and a methodology for detecting chloride-induced corrosion on-site, considering the difficulties associated with on-site assessment utilizing the polarization resistance method.

## Indirect methods

### Acoustic emission

One effective non-destructive method for detecting interior material deterioration is the acoustic emission (AE) approach. It is predicated on the identification of transient elastic waves that arise from localized changes in the material under test in response to an external load, surroundings, *etc.*^114,115^ The primary causes of emissions in reinforced concrete buildings include loading-induced microcracking and cracking in the concrete, as well as environmental factors. Piezoelectric transducers implanted in or affixed to the surface of the specimen under measurement receive the AE signal. Various AE signal characteristics, which are dependent on the kind of damage source or failure mechanism, are analyzed to evaluate the damage processes. A few examples of these parameters include counts, duration, energy, and amplitude. Preliminary studies suggest that the total number of hits could be a useful tool for locating the corrosion source and determining if the expansion of corrosion products has cracked the concrete cover.^116–118^ Furthermore, it has been demonstrated by Li *et al.*^116^ that corrosion beginning detection is possible before electrochemical approaches. They did not, however, provide a figure for the correlation between AE operations and the corrosion process. Additionally, recent research in ref. 119 provided findings on the correlation between the obtained AE signal strength parameters and the steel mass loss and concrete crack width. However, it's crucial to distinguish between the AE reaction brought on by corrosion and other outside stimuli like mechanical stresses, ambient noise from traffic, and surroundings. Further research is needed on this.

Many studies have conducted lab experiments on corrosion detection by using the AE method for different samples such as cubes,^120^ prism,^121^ beams^122^ and slabs.^123^ It was found that numerous AE methods (such as parameter analysis, cumulative rate, (*i*)*b*-value analysis, and moment-tensor analysis) have been conducted to produce consistent results; however, because they require the entirety of the history or place strict requirements on the quality and quantity of required data, they might be challenging to increase to on-site monitoring.

### On-site monitoring

Patil *et al.* did research^124^ on the detection of on-site corrosion using the acoustic emission (AE) method in conjunction with the half-cell electrochemical approach. The study determined that the AE methodology is a dependable strategy for assessing the probability of rebar corrosion and is well supported by electrochemical methods. The AE technique allows for the creation of a laboratory-based mathematical model that can be utilized to detect corrosion-induced damage in reinforced concrete (RC) structural components that are in service. This detection can be done without causing any harm to the element during testing. It is well accepted that monitoring structural components from the time they are first made is important for obtaining correct information on corrosion. However, this study shows that the acoustic emission (AE) technique is very effective in identifying damage caused by corrosion in buildings that are already in use. A shorter duration of continuous monitoring is sufficient for understanding the extent of corrosion in the materials under investigation. Hence, the use of the AE method may provide sufficient maintenance assistance and facilitate the implementation of suitable measures to effectively handle and prevent the structural failure of concrete structures.

### Optic fiber sensing (OFS)

In recent years, the optical fiber sensing technique has demonstrated considerable potential for structure health monitoring (SHM) on several grounds, including civil engineering. The advantages of this technology encompass exceptional accuracy, a small form factor, and resistance to disturbances like noise, corrosion, and electromagnetic interference, among several other attributes. An OFS comprises a fiber core, cladding, and a possible jacket or external coating that safeguards the fiber against mechanical and environmental strains. To direct the light waves as they propagate through the fiber, the light waves must undergo total internal reflection within the fiber, due to the cladding layer having a lower refractive index than the fiber core. Optical fiber sensors (OFSs) detect and interpret changes in light characteristics caused by changes in the item or environment being studied, allowing them to monitor external parameters. Several studies have suggested ways to invigilate the corrosion of the rebar in reinforced concrete structures, either by creating new OFSs^125^ or by utilizing OFSs that are already on the market.^126^

To create the innovative optical fiber sensors (OFSs), a unique coating material was deposited on the fiber, which has a controlled thickness. The coating material may degrade in the surrounding environment, leading to alterations in the optical properties that are crucial for sensing applications. The corrosion start time may be evaluated when the optical fiber sensor is mounted with its end positioned close to the depth of the embedded steel bars in new or present reinforced concrete (RC) structures. This is achieved by creating an optical fiber sensor (OFS) that incorporates an iron coating on the split end of the fiber. The decrease in the thickness of the iron layer in the corrosive environment leads to a decline in the intensity of reflected light, which is quantified. Additional investigation is required to establish a measurable relationship between the corrosion process and the alteration in reflectivity. It is important to note that this kind of OFS only provides information for the area in which it is placed.^125^

A sensing material composed of an iron–carbon layer placed along the fiber's grating area may be used to detect corrosion. As a result, new LPFG sensors for use in acidic conditions are born.^127^ LPFG, or long-period fiber gratings, are created by inserting gratings into the core of an optical fiber to induce a periodic modulation of the refractive index. At certain wavelengths, the grating period enables the transfer of light from the core mode to the cladding modes that propagate in the same direction. The grating period is larger than that of a fiber Bragg grating (FBG).^128^ The change in the resonance wavelength might be attributed to the corrosion-induced degradation of the outer Fe–C coating, resulting in a modification of the refractive index in that specific region. The experimental research examined the correlation between the resonant wavelength changes and the corrosion penetration depth of the detecting Fe–C coating. These experiments utilized both naked optical fiber sensors (OFS) and OFS^129^ implanted in concrete. By imprinting a single fiber with a small number of grating areas that have varying grating periods and refractive indices, quasi-distributed sensing may be accomplished. Still, there is a subset of sensors known as LPFG sensors that can only measure corrosion reactions inside the grating zone. An often-asked concern about new oxide film systems (OFSs) is the extent to which the corrosion behavior of the iron (Fe) or iron–carbon (Fe–C) coating layer on OFS can accurately replicate the corrosion condition of the rebar. Despite having the same material composition and being exposed to the same environmental conditions, the corrosion characteristics of the film and steel rebar might differ due to their unique microstructures and sizes. Nevertheless, there has been no previous investigation of this matter.

Commercially accessible optical fiber sensors (OFSs), including distributed optical fiber sensors (DOFS) and fiber Bragg gratings (FBG), can be affixed to the concrete surface to directly monitor the stresses caused by corrosion-induced defects on the steel rebar surface. The Bragg wavelength shift, influenced by fluctuations in strain or temperature at the sensor's position, can be utilized for the detection and monitoring of corrosion.^130^ Due to the strong correlation between temperature and the temperature of the fiber, Raman scattering is frequently employed for temperature sensing. In contrast, Brillouin and Rayleigh scattering may be utilized to detect both strain and temperature. It is shown that optical frequency domain reflectometry (OFDR) may potentially attain a much greater spatial resolution, reaching the sub-millimeter level, when contrasted with Brillouin scattering analysis and Rayleigh scattering analysis.^131^ This approach is valuable for assessing the magnitude and precise localization of corrosion-related issues.

**Fig. 14:**
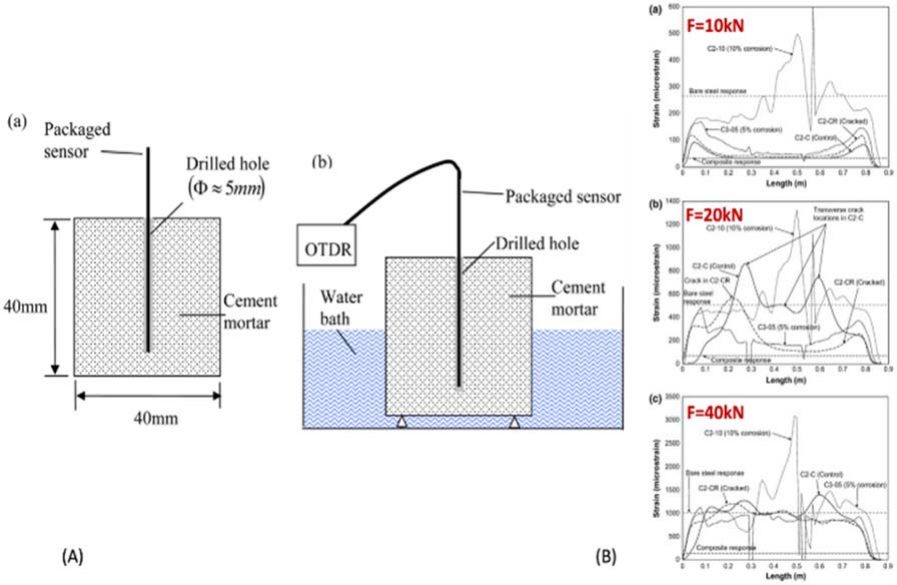
(A) Graphical illustration of experimental setup for corrosion detection using optical fiber sensor, (B) results of corroded rebars using by optical fiber under different applied loads.

In laboratory experiments, FBG and DOFS have been used to track strain and cracking in concrete caused by corrosion.^132^ As corrosion products accumulate, the pressure within the concrete around the steel cross-section increases, causing hoop tensile strain. This leads to twisting of the concrete around the surface of the rebar. Additionally, DOFS which is known as distributed OFS has been utilized along the lateral direction of the steel to measure the strain of corroded rebar underloaded externally. This has allowed researchers to link bond degradation to ref. 133 and pitting corrosion level to ref. 126, as well as to associate strain localization with ref. 134. The DOFS measurement was used to track the strain fluctuation along the corroded rebar in an RC beam,^135^ which is depicted in Fig. 14(B) below with applied loads of 10 kN, 20 kN, and 40 kN, while Fig. 14(A) shows the illustrative sketch of the lab experimental setup for the optical fiber sensing method to detect the corrosion level.

### On-site monitoring

While using optical fiber sensing (OFSs) on the rebar surface in concrete structures has shown encouraging results, there are several problems with real-world implementations. The first problem is that to allow monitoring for a longer period, OFSs require strong bonding and protection in concrete. However, because of the shear lag effect, the protective layer and bonding agent may impede the measurement of strain, making it impossible to accurately reflect the true strain on the steel. Second, in addition to pitting corrosion, shrinkage cracking and flexural cracking in concrete can also affect the stress distribution along the steel rebar. Finding a way to differentiate between them will be a crucial topic for getting accurate quantitative findings. In a study^136^ optical fibers were used to evaluate the structural capacity of existing bridges. However, there are fewer studies on the chloride corrosion detection of existing structures by optical fiber sensing methodology.

**Table 1:** Summarizes the working principle along with the advantages and limitations of corrosion detection techniques discussed in this review paper

Method	Principle	Lab advantages	Lab limitations	*In situ* advantages	*In situ* limitations
Electromagnetic	Detects changes in magnetic properties due to corrosion	Detects corrosion zones, links response to steel loss	Alignment of corrosion and magnetic flux affects success	Localizes corrosion zones	Blockage of magnetic flux by corrosion layers may occur
3D laser scanning	Generates 3D visual representation of rebar surface	Accurate measurement of pit depth, steel mass loss	Destructive (rebar must be removed from concrete)	Not typically used *in situ* due to removal requirements	Cannot monitor the same sample at different intervals
X-ray micro-CT	3D examination of internal structures using X-rays	High-resolution tracking of corrosion progression	Limited to small samples, high cost	Tracks corrosion progression and internal damage over time	Requires small samples to be removed for lab analysis
Electrochemical methods	Measures corrosion rate by analyzing electrochemical signals	Quantifies corrosion accurately, well established in labs	Complex setup, affected by environmental conditions	Direct measurement of corrosion rates	Precision is limited in the field due to an unknown polarized area
Acoustic emission (AE)	Detects transient elastic waves caused by internal damage	Early detection distinguishes corrosion from other damages	Difficult to differentiate AE signals from external noise	Suitable for monitoring service structures	Requires continuous monitoring, high sensitivity to external noise
Optical fiber sensing	Detects strain and deformation through changes in light transmission	High accuracy, measures strain and deformation	Requires strong bonding, complex setup	Continuous monitoring of corrosion	Difficult to differentiate between strain from corrosion and other stresses

## Conclusion

• Qualitative non-destructive methods, such as half-cell potential (HCP) or concrete resistivity tests, are frequently employed in practice for corrosion evaluation of in-service reinforced concrete structures. Half-cell potential measurement only provides the likelihood of corrosion at a certain location and time and is affected by numerous factors such as the cathode-to-anode ratio, the availability of oxygen near the steel surface, the composition of the pore solution (pH, chloride or sulphide content), and the geometry, resistivity, cover depth, and presence of cracks in the concrete. Table 1 provides an brief summary of the advantages and disadvantages of the methods used for corrosion detection in the lab as well as in the field.

• Electrochemical methods such as the Tafel extrapolation technique, LPR, or AC impedance spectroscopy are used to quantify corrosion by estimating the corroded condition from the received electrical data. These methods are typically thought to be able to detect the corrosion process when it has progressed to an advanced level and estimate the instantaneous corrosion rates that correspond to the test circumstances at that moment.

• The rate of corrosion varies depending on temperature, the resistivity of the concrete, the availability of oxygen, *etc.* As a result, it is challenging to extrapolate service life from a single test, and to understand the average corrosion rate over a longer time, many observations under various seasonal circumstances are needed. During the test time, this can cause changes to the system.

• Furthermore, the existing structures cannot feasibly accommodate these electrochemical approaches since they necessitate either electrical or physical interaction with steel embedded in concrete.

• Non-destructive methods such as AE, optical fiber sensing, *etc.* are a good choice in evaluating the corrosion level at any time in the lab or on-site. However, these methods require strong technical skills for the post-processing of the data. Second, it is a bit expensive to evaluate an existing structure. In case the availability of such systems is made easier still need strong technical knowledge to post-process the data.

## Future recommendation

Traditional methods and techniques for measuring chloride-induced corrosion struggle with the outcomes, some provide quantitative analyses while the rest provide the corrosion rate, *etc.* To overcome these issues latest structural health monitoring (SHM) techniques such as 3D laser scanning, X-ray μCT, and optical fiber sensing techniques have been developed and these methods have been proven more effective than HCP, concrete resistivity tests and other electrochemical methods in the detection of chloride-induced corrosion. The factors that make these methods superior are the lesser effect of environmental conditions on the results and are also independent of the level of corrosion, time of detection of corrosion, *etc.* However, these methods have some drawbacks such as being expensive, the limitation of the sample to be taken to the lab, *etc.* With the recent advancement in SHM in the civil engineering field the use of AI tools has also been expanded. CNN models have been a great tool recently in SHM techniques for damage detection. Different image processing techniques of CNN models have been employed accurately for the monitoring of structures. The authors have found a clear gap in the use of AI tools such as CNN and ANN models in the detection of chloride-induced corrosion. Further studies on chloride-induced corrosion detection must be conducted with AI tools and 3D laser scanning, X-ray μCT, and optical fiber sensing techniques. The models should be developed to analyze the image received from these techniques which may lower the cost and time spent on these methods.

## Data availability

No data was used for the research described in the article. This study is based on theoretical analysis/literature review and does not involve any experimental or primary data.

## Author contributions

MA: conceptualization; data curation; methodology; writing-original draft. MAS: conceptualization; software; data curation; methodology; writing-original draft; writing-review & editing. NB: data curation; methodology; visualization; writing-original draft; writing-review & editing. A. H. A.: formal analysis; methodology; visualization; writing-review & editing. A. S. M.: formal analysis; software; visualization; writing-review & editing. Y. A. D.: conceptualization; visualization; writing-review & editing. O. B.: formal analysis; visualization; validation; writing-review & editing.

## Conflicts of interest

There are no conflicts to declare.
